# Modulatory effects of magnetic vestibular stimulation on resting-state networks can be explained by subject-specific orientation of inner-ear anatomy in the MR static magnetic field

**DOI:** 10.1007/s00415-020-09957-3

**Published:** 2020-06-11

**Authors:** R. Boegle, V. Kirsch, J. Gerb, M. Dieterich

**Affiliations:** 1 Department of Neurology, University Hospital, Ludwig-Maximilians-Universität München, Marchioninistraße 15, 81377 Munich, Germany; 2 Graduate School of Systemic Neuroscience (GSN), Ludwig-Maximilians-Universität, Munich, Germany; 3 German Center for Vertigo and Balance Disorders DSGZ-IFB LMU, University Hospital, Ludwig-Maximilians-Universität, Munich, Germany; 4 Munich Cluster for Systems Neurology (Synergy), Munich, Germany

**Keywords:** Magnetic vestibular stimulation, MVS, MRI static magnetic field stimulation, MRI B0 magnetic field, fMRI, Resting state, RS-fMRI, Vestibular system, Inner ear, Laterality, Lateralization

## Abstract

Strong static magnetic fields, as used in magnetic resonance imaging (MRI), stimulate the vestibular inner ear leading to a state of imbalance within the vestibular system that causes nystagmus. This magnetic vestibular stimulation (MVS) also modulates fluctuations of resting-state functional MRI (RS-fMRI) networks. MVS can be explained by a Lorentz force model, indicating that MVS is the result of the interaction of the static magnetic field strength and direction (called “B0 magnetic field” in MRI) with the inner ear’s continuous endolymphatic ionic current. However, the high variability between subjects receiving MVS (measured as nystagmus slow-phase velocity and RS-fMRI amplitude modulations) despite matching head position, remains to be explained. Furthermore, within the imaging community, an “easy-to-acquire-and-use” proxy accounting for modulatory MVS effects in RS-fMRI fluctuations is needed. The present study uses MRI data of 60 healthy volunteers to examine the relationship between RS-fMRI fluctuations and the individual orientation of inner-ear anatomy within the static magnetic field of the MRI. The individual inner-ear anatomy and orientation were assessed via high-resolution anatomical CISS images and related to fluctuations of RS-fMRI networks previously associated with MVS. More specifically, we used a subject-specific proxy for MVS (pMVS) that corresponds to the orientation of the individual inner-ear anatomy within the static magnetic field direction (also called “*z*-direction” in MR imaging). We found that pMVS explained a considerable fraction of the total variance in RS-fMRI fluctuations (for instance, from 11% in the right cerebellum up to 36% in the cerebellar vermis). In addition to pMVS, we examined the angle of Reid’s plane, as determined from anatomical imaging as an alternative and found that this angle (with the same sinus transformation as for pMVS) explained considerably less variance, e.g., from 2 to 16%. In our opinion, an excess variability due to MVS should generally be addressed in fMRI research analogous to nuisance regression for movement, pulsation, and respiration effects. We suggest using the pMVS parameter to deal with modulations of RS-fMRI fluctuations due to MVS. MVS-induced variance can easily be accounted by using high-resolution anatomical imaging of the inner ear and including the proposed pMVS parameter in fMRI group-level analysis.

## Introduction

Strong magnetic fields exceeding 1 T are commonly used in magnetic resonance imaging (MRI) and were demonstrated to stimulate the vestibular system [1–5]. This magnetic vestibular stimulation (MVS) leads to a state of vestibular imbalance, which is verifiable in nystagmus [1–3] and modulations of resting-state (RS) networks fluctuations via the stimulation of vestibular brain areas [4, 5]. The proposed mechanism for MVS is based on a Lorentz force model that explains not only the occurrence, direction, and persistence of the observed nystagmus, but also its dependence on the head positions within the static magnetic field (also called “B0 magnetic field” in MRI) and the strength of the magnetic field itself [1–7].

Unexplained so far is that associated measures of MVS, such as the nystagmus slow-phase velocity and the RS-fMRI fluctuations, show a considerable between-subject variance [1–4]. This between-subject variance increases linearly with field strength even if subjects were all measured in the same head position [1, 4]. In the framework of the Lorentz force model, these differences between subjects can be explained by the remaining factors: the current flow (density of potassium ions in the inner ear) and the direction of the flow relative to the magnetic field (i.e., the morphology of the inner ear with varying angles of the horizontal semicircular canals) [1, 3]. Other potential covariates that are not covered by the Lorentz force model could be the subject’s alertness or attention level or the general level of individual excitability of the subject’s vestibular system.

So far, only additional video-oculography (VOG) equipment or elaborate simultaneous VOG/fMRI measurements can help estimate MVS influence on fMRI [4]. However, when considering both future tendencies to use higher magnetic fields and the ubiquitous nature of MVS in any functional MRI study, such a proxy will be highly sought after within the imaging community.

In this study, our primary aim was to find an “easy-to-acquire-and-use” proxy parameter (pMVS) that mirrors the orientation of the individual inner-ear anatomy within the static magnetic field that can account for modulatory MVS in RS-fMRI fluctuations. We, therefore, focused on between-subject variability in RS-fMRI data from 60 healthy volunteers and its correlates within high-resolution inner-ear anatomical images to examine pMVS modulatory effects on RS-fMRI fluctuations in six regions known to be influenced by MVS [4, 5]. Other non-imaging (psychological) cofactors were left for future studies.

## Materials and methods

The data used in this study were partly published previously [8, 9]. The data on the morphology of the inner ear are unpublished data.

### Participants

Institutional Review Board (IRB) approval was obtained before the initiation of the study. Each participant provided informed oral and written consent following the Declaration of Helsinki. Sixty (60) healthy volunteers balanced for age, gender, and handedness were initially included in the study. However, five had no CISS images of the inner ear (see below) and were therefore not used in the analysis, which leaves a total of 55 participants (26 males (aged 20–65 years, mean age 26.1 ± 8.6; 12 right handers, 14 left handers) and 29 females (aged 20–67 years, mean age 26.7 ± 8.3; 13 right handers, 16 left handers) in the final analyses. The handedness laterality quotient was assessed with the ten-item inventory of the Edinburgh test [10, 11]. The laterality quotient for right-handedness was + 100% in all 25 right-handers. The laterality quotient for left-handedness was − 100% in 15, between – 90 and − 80% in 12 and between − 70 and − 65% in three of the 30 left-handed volunteers.

The inclusion criterion was age between 18 and 70 years. Exclusion criteria were a history of any neurological, vestibular, and/ or psychiatric disorder, pregnancy, and MR-related contraindications, such as cardiac pacemakers, ferromagnetic implants, or claustrophobia.

### Measurement of the semicircular canal and otolith functions

The integrity of the vestibular function was ascertained by assessing the semicircular canal function with the video head-impulse test for the vestibulo-ocular reflex (VOR) and with the determination of the subjective visual vertical (SVV) for otolith function, respectively. The head-impulse test [10] was measured using high-frame-rate video-oculography (VOG) with EyeSeeCam ([11]; EyeSeeTech, Munich, Germany). A median gain during head impulses below 0.8 (eye velocity in °/s divided by head velocity in °/s) was considered the criterion for a pathological VOR and exclusion of the subject. As the tilt of the SVV is a sensitive sign of a graviceptive vestibular tone imbalance, we considered a mean deviation higher than ± 2.5° from the true vertical as pathological. SVV was assessed while sitting in an upright position in front of a half-spherical dome with the head fixed on a chin rest (for details see [12]). None of the participants showed deficits in the two measures.

### Imaging protocol

MR imaging data were acquired in a whole-body 3.0 T MR scanner (Magnetom Verio, Siemens Healthcare, Erlangen, Germany) with a 32-channel head coil. Intrinsic brain activity was assessed with BOLD fMRI based on T2*-weighted echo-planar imaging (EPI) sequence with a 3.0 × 3.0 × 3.0 mm^3^ isotropic resolution (TE = 30 ms, TR = 3000 ms, 200 frames per subject). No other task was required except keeping the eyes closed, remaining still without focusing the thoughts on anything specific, and not falling asleep. The time between “being inside the scanner” and the “start of RS-fMRI measurement” varied over all subjects; however, all subjects, but one (1), were in the MRI scanner for at least 5 min before the RS-fMRI sequence was started. This time was included in the statistical analysis, see the section below. Anatomical images included a T1-weighted magnetization-prepared rapid gradient echo (MP-RAGE) sequence with a field-of-view of 256 mm and an isotropic spatial resolution of 1.0 × 1.0 × 1.0 mm^3^ (TE 4.37 ms, TR = 2100 ms, number of slices 160). Furthermore, a high-resolution, strongly T2-weighted, 3D constructive interference steady state (CISS) sequence of the temporal bones was planned with regards to the T2-weighted sequence. CISS sequence was performed to assess the individual anatomy of the inner ear and its spatial alignment within the magnetic field (the angle between the horizontal semicircular canal and static magnetic field direction). The following parameters were used: TR 1000 ms, TE 138 ms, FoV phase 100°, FOV read 180 × 180 mm^2^, 60 slices, base resolution 384, averages 2, slice thickness of 0.5 mm. To control for potential head movement in the pitch axis between the acquisition of the CISS sequence and the BOLD data, a “CRANIA adult” inflatable head cushion, was used to keep the subjects head movement to a minimum. Overall, head motion measured by the mean relative displacement (in mm) was low (0.09 ± 0.13). Also, the co-registration of the T2- and T1-weighted sequence with the RS-fMRI sequence was checked. None of the datasets showed changes in the pitch axis. Five participants lacked a CISS sequence and were therefore excluded from further analyses. The following analyses were done with the remaining 55 participants.

### fMRI data processing

All acquired images in DICOM format were converted to the NifTI-file format (using MRICron dcm2nii) and preprocessed using the SPM12 software package, DARTEL (Diffeomorphic Anatomical Registration Through Exponentiated Lie algebra; [13, 14]) and CONN functional connectivity toolbox v15 [15]. Further denoising using whole-brain ICA as well as dual regression as implemented in the FSL toolbox MELODIC [16, 17], and scripts in MATLAB (MathWorks Inc., Natick, MA, USA) were applied as previously described in [8, 9, 18]. No participants were discarded due to motion artifacts.

Associated codes and maps can be viewed and downloaded from GitHub (“https://github.com/RainerBoegle/BeyondBinaryParcellationData”, “https://github.com/RainerBoegle/DeNoiseFromDualReg”).

Following preprocessing, an 80-dimensional whole-brain group ICA was performed on the combined datasets [16]. The dimensionality of the ICA was estimated by the FSL MELODIC default method [16]. All independent component (IC) maps were thresholded at *p* > 0.5 for the alternative hypothesis test using the Gaussian/gamma mixture model approach implemented in MELODIC [16]. Then, amplitude maps for all components and all subjects were created using dual regression. In the first step, time courses were obtained by regressing the group independent component maps on the fMRI data of each subject. These time courses were used in the second step to create amplitude maps via regression of the normalized time courses (demean and scaling by standard deviation over time) back on the fMRI data of each subject, thus resulting in response amplitudes at every voxel (and time courses) for each subject [17, 19].

### Building a subject-specific proxy to account for MVS (or pMVS)

**Fig. 1:**
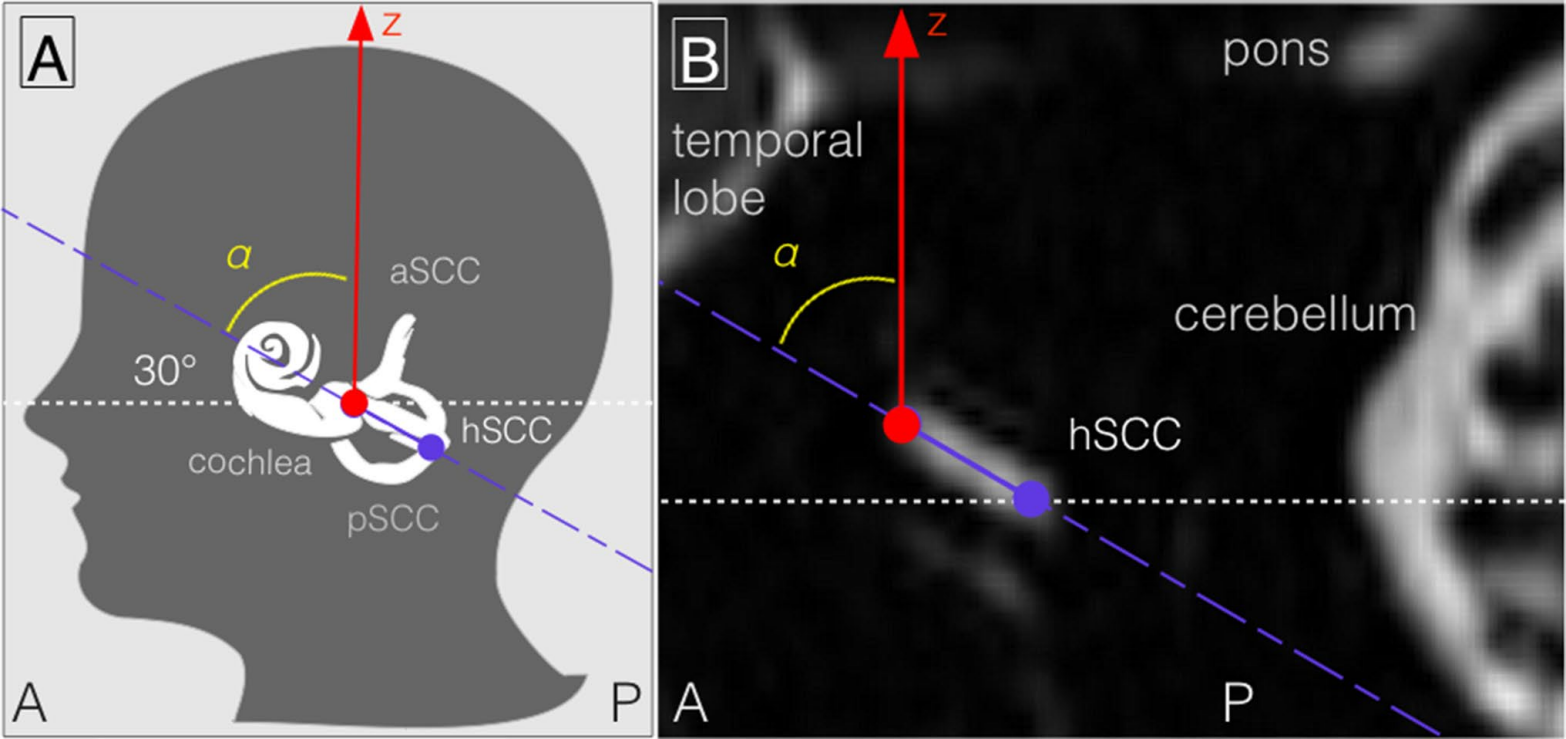
**a** Schematic drawing to depict the assessment of the individual anatomy of the inner ear and its subject-specific spatial alignment within the magnetic field (also called “B0 magnetic field”). A subject-specific proxy to account for MVS (pMVS) mirrored the orientation of the individual inner-ear anatomy within the static magnetic field direction (also called “*z*-direction”). pMVS was defined as the sinus of angle *α* ($\sin (\alpha$)) between the positional plane running through the horizontal semicircular canal (hSCC in purple) and the static magnetic field direction (labeled as “z” and marked in red). **b** Exemplary CISS image after being reconstructed in the sagittal plane to simplify the assessment of angle *α.* In consequence, that magnetic field direction was perpendicular to the upper limit of the frame, i.e., the z-direction was upwards. The angle could then be easily measured between the line of the positional plane running through the horizontal semicircular canal (marked in purple) and magnetic field direction *z* (= the upper frame’s perpendicular, marked in red). Following (**a**) labels “A” and “P” mark the front (*A* anterior) and back (*P* posterior) of the head. Further abbreviations: *aSCC* anterior semicircular canal, *pSCC* posterior semicircular canal

The individual anatomy of the inner ear was assessed using the high-resolution CISS sequences of the temporal bones. The subject-specific proxy to account for MVS (pMVS) was designed to mirror the spatial orientation of the individual inner-ear anatomy within the static magnetic field direction (also called “*z-*direction”) which was defined as the sinus of the angle *α* between the positional plane of the horizontal semicircular canal (hSCC) and the static magnetic field direction “*z*-direction”. To simplify the assessment, the high-resolution CISS data were reconstructed in the sagittal plane in such a way that the static magnetic field direction (“*z*-direction” in MRI) was perpendicular to the upper limit of the frame, i.e., the “*z*-direction” was upwards in the image (see Fig. 1a). The angle could then be easily measured between the positional plane running through the horizontal semicircular canal (see Fig. 1b) and the *z*-direction (the upper frame’s perpendicular). The angles of the left and right side were measured, and the mean of both angles used for further analyses. Furthermore, these angles were compared to Reid’s plane. Reid’s plane is the line between the infraorbital margin of the orbita and the upper margin of the external auditory meatus.

### Regions of interest

Six regions of interest (ROIs) were selected based on results from two previous studies [4, 5] as well as symmetry considerations, as the vestibular system has distinct lateralization properties related to handedness [4, 5, 8, 9, 20, 21]. The two previous MVS studies [4, 5] had found MVS modulations in the default mode network (DMN) and a visual network called the “higher visual network” (hVN) in the scaling relationship of RS-fMRI fluctuations in 1.5 T and 3.0 T. Here these ROIs serve as areas for testing whether RS-fMRI fluctuations are dependent on the different orientations of the inner ears, as these areas were previously found to be MVS sensitive.

**Fig. 2:**
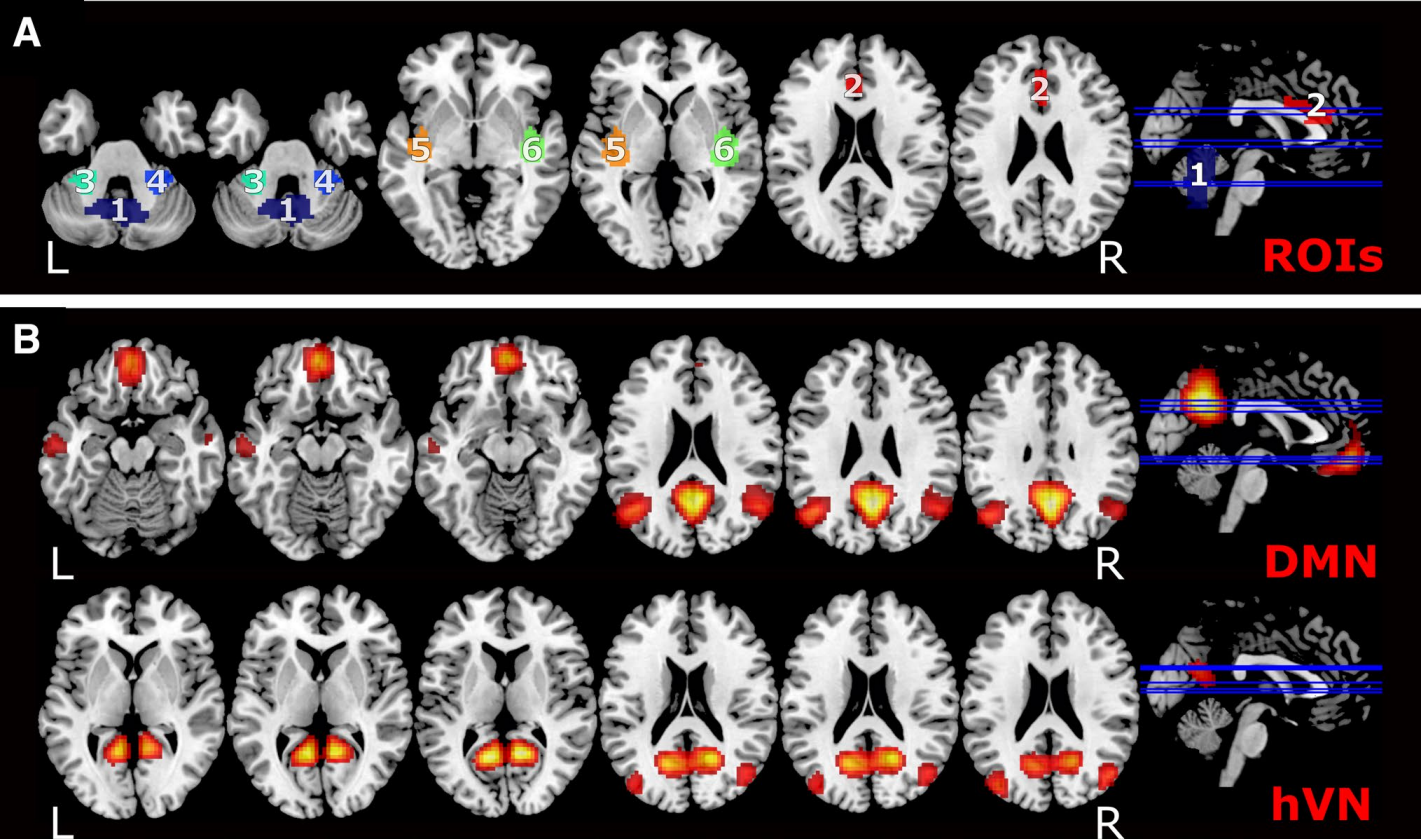
(A) Overlay of the six regions of interest (ROIs) used in the analysis. ROIs were numbered (1–6), colored, and projected onto an anatomical brain template. ROI 1 is situated in the cerebellar vermis, ROI 2, in the anterior cingulum, both along the midline. ROI 1 and ROI 2 were taken from the DMN of a previous MVS study [4]. ROI 3 and 4 are located on the left and right side of the cerebellum, respectively. ROI 5 and 6 can be found in the left and right posterior insula. ROI 3 and ROI 6 were taken from the hVN as in a previous study [5]. The two previous MVS studies [4, 5] had found MVS modulations in the default mode network (DMN) and a visual network called the “higher visual network” (hVN) in the scaling relationship of RS-fMRI fluctuations in 1.5 T and 3.0 T. ROI 4 and ROI 5 are the respective mirror sites of ROI 3 and ROI 6. (B) Overlay of the resting-state networks of interest (RSNs): The default mode network (DMN) can be viewed in the upper and the “higher visual network” (“hVN”) in the lower row. The RSN is shown superimposed on an anatomical brain template. Further abbreviations: L = left, R = right)

Two regions from the DMN were situated in the cerebellar vermis and the anterior cingulate cortex, i.e., areas along the midline. Two other regions from the “higher visual network” were in the right posterior insula and the left cerebellar hemisphere. For symmetry considerations, these two lateralized regions were turned into four areas by also including the mirrored versions of these two areas (see Fig. 2). The chosen ROIs were

- ROI 1 “cerebellar vermis” [mean MNI (0 mm, − 60 mm, − 27 mm)],
- ROI 2 in the anterior cingulate cortex [mean MNI (0 mm, 27 mm, 23 mm)],
- ROI 3 covering a region in the left cerebellar hemisphere [mean MNI (− 24 mm, − 39 mm, − 30 mm)],
- ROI 4 the same region in the right cerebellar hemisphere [mean MNI (30 mm, − 39 mm, − 33 mm)], corresponding to cerebellar lobule V and VI. Furthermore,
- ROI 5 included the left posterior insula [mean MNI [− 39 mm, − 15 mm, 0 mm)] and
- ROI 6 the right posterior insula [mean MNI (42 mm, − 15 mm, 0 mm)].

ROI 5 and 6 covered area Id1, Ig2, and reached into the superior temporal gyrus. All ROIs are depicted in Fig. 2a.

### Statistics and map display

ROI-specific amplitudes were averaged over space separately (i.e., all voxels in each ROI, were averaged using the arithmetic mean of all values) and analyzed via a general linear model for group effects with regressors for constant (sex and handedness) and continuous covariates (inner-ear parameters, angle of Reid’s plane, age, time since entering the MRI).

Regression coefficients *β* (slopes) and residuals were estimated using the robust regression function “*robustfit.m*” in MATLAB. In addition, the standard error of regression (SE) for each beta (slope) was determined in each ROI as output by “*robustfit.m*” (see Fig. 4 for the betas and standard errors; see Fig. 5 for the *R*^2^ values). The included continuous and constant regressors were:

- “pMVS”: a subject-specific proxy for MVS (continuous value regressor),
- “pReidsAv”: alternatively to “pMVS”, we used the sinus-transformed (analog to pMVS) angle of Reid’s plane in the regression, determined as described above.
- “Age”: age of each subject in years (continuous value regressor),
- “ranks(Age)”: age of each subject replaced by the rank among all subjects using MATLAB’s “tiedrank.m”: function (continuous value regressor),
- “dt-RSfMRI”: the time between the start of the first sequence in the MRI session of each participant and the start of the RS-fMRI sequence in minutes (continuous value regressor),
- “ranks(dt-RSfMRI)”: analog to above, the replacement of each time with the rank among all times of all participants (a continuous value regressor),
- “Sex: female”: female participants (a constant regressor),
- “Sex: male”: male participants (a constant regressor),
- “Handedness RH”: all right-handed participants (a constant regressor) and
- “Handedness LH”: all left-handed participants (a constant regressor).

The regressor (or the subject-specific proxy to account for MVS, pMVS) was calculated by transforming the measured angles “*α*” of the horizontal canal relative to the z-direction (or magnetic field direction) to radians and then determining the sinus of “*α*”, as required for the Lorentz force: $\vec{F} = \vec{I} \times \overrightarrow {B } = \left| I \right| \cdot \left| B \right| \cdot \sin \alpha$. In summary, this resulted in a design matrix with nine regressors (one regressor for pMVS or pReidsAv, and the other eight regressors).

As a next step, these “continuous” regressors and ROI data were *z*-scored using MATLAB’s “*z*score.m” function before fitting the slopes (betas). This adjustment of the regressors and the data resulted in “normalized” slope (beta) values. Before plotting the data and regression line (slope) for pMVS, we removed the effects of all other regressors (age, time until RS-fMRI measurement, sex, and handedness) by means of subtraction from the data to keep only the pMVS effects (and remaining other variance) for display.

To understand the contribution of the regressors for sex, handedness, and pMVS more closely, we used a stepwise regression approach, which enables the estimation of the effect size in terms of R-squared (coefficient of determination) added by each variable following the recommendations in [22, 23]. In short, we created all possible permutations of the variables in the regression model and repeatedly applied the regression adding one more regressor at each step. Each of the steps for each permutation results in an *R*-squared value (% of explained variance). Then, the added R-squared effect size was estimated as the median of all differences in *R*-squared for each regressor in all models for every permutation. In other words, the added *R*-square in each step was the difference of the *R*-square value for that step to the previous step, and the final added *R*-squared value (% of explained variance) for each variable was estimated as the median of these differences. The final added *R*-squared value for each variable, and each ROI was reported separately in Table 1 and displayed in Fig. 5.

**Table 1:** This table focuses on between-subject variability in RS-fMRI data, as analyzed with two models, one with pMVS and one with pReidsAv as the main covariate of interest, and all other covariates of no interest, like age, sex, handedness and dt-RSfMRI being the same in both models

	Cerebellar vermis	Anterior cingulum	Cerebellum left	Cerebellum right	Posterior insula left	Posterior insula right
Regressor	Beta ± SE					
pMVS pReidsAv	** + 0.77 ± 0.1** ** + 0.29 ± 0.1**	** + 0.32 ± 0.2** ** + 0.06 ± 0.1**	**− 0.47 ± 0.1** **− 0.17 ± 0.1**	** + 0.28 ± 0.2** ** + 0.34 ± 0.2**	**− 0.42 ± 0.2** **− 0.17 ± 0.1**	**− 0.47 ± 0.2** **− 0.41 ± 0.1**
Sex: female	− 0.27 ± 0.2 + 0.17 ± 0.3	** + 0.5 ± 0.3** ** + 0.8 ± 0.3**	− 0.01 ± 0.2 − 0.13 ± 0.3	+ 0.25 ± 0.3 + 0.18 ± 0.3	− 0.07 ± 0.3 − 0.15 ± 0.3	+ 0.03 ± 0.3 + 0.07 ± 0.3
Sex: male	− 0.03 ± 0.1 − 0.08 ± 0.2	− 0.02 ± 0.2 − 0.00 ± 0.2	− 0.00 ± 0.1 + 0.00 ± 0.1	+ 0.02 ± 0.2 + 0.00 ± 0.2	+ 0.00 ± 0.2 − 0.01 ± 0.2	+ 0.02 ± 0.2 + 0.01 ± 0.2
RH	+ 0.33 ± 0.2 − 0.24 ± 0.3	**− 0.45 ± 0.3** **− 0.69 ± 0.3**	− 0.29 ± 0.2 + 0.01 ± 0.3	− 0.23 ± 0.3 − 0.33 ± 0.3	− 0.06 ± 0.3 + 0.11 ± 0.3	− 0.08 ± 0.3 − 0.06 ± 0.3
LH	− 0.03 ± 0.2 − 0.09 ± 0.2	− 0.08 ± 0.2 − 0.25 ± 0.2	+ 0.10 ± 0.2 − 0.00 ± 0.2	− 0.03 ± 0.2 + 0.10 ± 0.3	+ 0.01 ± 0.2 − 0.11 ± 0.2	+ 0.08 ± 0.2 − 0.10 ± 0.2
Age	+ 0.08 ± 0.1 − 0.52 ± 0.4	− 0.08 ± 0.2 − 0.52 ± 0.4	− 0.04 ± 0.1 − 0.15 ± 0.4	− 0.16 ± 0.2 − 0.34 ± 0.4	+ 0.07 ± 0.2 − 0.22 ± 0.4	+ 0.09 ± 0.2 − 0.21 ± 0.4
Ranks*	− 0.39 ± 0.1 − 0.10 ± 0.2	− 0.04 ± 0.2 + 0.13 ± 0.2	+ 0.21 ± 0.1 + 0.22 ± 0.2	+ 0.22 ± 0.2 + 0.34 ± 0.2	+ 0.02 ± 0.2 + 0.12 ± 0.2	+ 0.17 ± 0.2 + 0.24 ± 0.2
dt-RSfMRI	− 0.24 ± 0.2 − 0.35 ± 0.2	+ 0.24 ± 0.2 + 0.06 ± 0.2	− 0.16 ± 0.2 − 0.15 ± 0.2	+ 0.03 ± 0.2 − 0.04 ± 0.3	+ 0.08 ± 0.2 + 0.14 ± 0.3	+ 0.14 ± 0.2 + 0.20 ± 0.3
Ranks*	+ 0.06 ± 0.2 + 0.22 ± 0.2	− 0.16 ± 0.2 + 0.06 ± 0.2	+ 0.07 ± 0.2 + 0.07 ± 0.2	+ 0.06 ± 0.2 + 0.11 ± 0.3	+ 0.00 ± 0.2 − 0.07 ± 0.3	+ 0.02 ± 0.2 − 0.08 ± 0.3
Regressor	*p* value					
pMVS pReids	**6e-8** **0.049**	**0.046** **0.671**	**6e-4** **0.198**	**0.081** **0.029**	**0.008** **0.245**	**0.005** **0.007**
Sex: female	0.209|0.561	**0.077 | 0.006**	0.976|0.619	0.378|0.553	0.795|0.603	0.915|0.823
Sex: male	0.816|0.664	0.927|0.983	0.989|0.971	0.921|0.982	0.993|0.942	0.890|0.960
RH	0.119|0.369	**0.100|0.011**	0.196|0.982	0.409|0.243	0.808|0.680	0.762|0.826
LH	0.861|0.696	0.702|0.293	0.600|0.998	0.904|0.681	0.979|0.655	0.714|0.692
Age	0.566|0.198	0.634|0.188	0.791|0.683	0.373|0.419	0.667|0.606	0.628|0.617
Ranks*	**0.005|0.661**	0.799|0.587	0.154|0.320	0.203|0.175	0.929|0.623	0.340|0.333
dt-RSfMRI	0.183|0.161	0.305|0.799	0.421|0.525	0.912|0.875	0.736|0.597	0.563|0.434
Ranks*	0.737|0.375	0.475|0.808	0.714|0.757	0.798|0.668	0.986|0.764	0.926|0.753
Variable	%-Explained variance					
pMVS pReidsAv	**35.7%** **16.3%**	**13.9%** **5.9%**	**26.1%** **2.4%**	**11.0%** **13.0%**	**18.1%** **3.4%**	**13.3%** **11.2%**
Sex	2.3% 2.1%	**8.1%** **16.3%**	0.5% 1.9%	2.8% 3.3%	2.1% 1.8%	1.4% 1.1%
Handedness	1.7% 1.0%	**2.0%** **4.0%**	2.2% 0.1%	1.0% 2.6%	0.6% 1.4%	0.1% 0.3%
Age	**11.9%** **18.4%**	3.3% 7.6%	5.5% 6.5%	3.1% 3.5%	2.0% 3.7%	7.2% 6.0%
dt-RSfMRI	3.7% 3.3%	1.0% 1.8%	3.7% 2.4%	1.1% 2.2%	1.0% 1.2%	1.1% 0.8%

Here, regression slopes (betas) with standard error of regression (SE) can be found on top, probability values for the test of the betas (H0: beta is zero, i.e., slope if flat) in the middle and the median (across all permutations) amount of explained variance in percent for the stepwise regression model on the bottom. The significance level for the six regions of interest was 0.0083 (= 0.05/6). All variables for each region that showed (single) significant results were marked in bold. In the (top) and (middle) parts, “rank*” was used to represent the ranked version of the regressor above (either “Age” or “dt-RSfMRI”). The variable “dt-RSfMRI” represents the time (in minutes) that has passed from the beginning of the first MRI sequence until the beginning of the RS-fMRI sequence

Then, the differences between the pMVS regressor slope for the left and right cerebellar regions (ROIs 3 and 4) as well as the left and right posterior insula regions (ROIs 5 and 6) were tested using the approach described in the Appendix “Measurement of the semicircular canal and otolith functions” of [24]. The test statistic is *t*-distributed with $t = \left( {\beta_{2} - \beta_{1} } \right)/\sqrt {{\text{SE}}_{\beta 1}^{2} + {\text{SE}}_{\beta 2}^{2} }$, with ${ }\beta_{1}$ and $\beta_{2}$ being the beta (slope) values of the pMVS regressors of the respective ROIs and ${\text{SE}}_{\beta 1}$ and ${\text{SE}}_{\beta 1}$ being the standard errors of regression for $\beta_{1}$ and $\beta_{2}$, respectively. Effective degrees of freedom were *df* = N − 4 = 51 (four parameters: two betas and two standard errors have to be estimated; therefore, these degrees of freedom have to be removed).

The script and data for the complete statistical analysis of the mean RS-fMRI data in each ROI with the mentioned covariates will be available at https://github.com/RainerBoegle/MVSinnerEarCorr.

## Results

In brief, the proxy for the subject-specific morphology of the inner ear (pMVS) showed significant modulations of RS-fMRI amplitudes in four out of the six chosen ROIs, albeit with a different direction, effect strength, and explained variance. For five out of the six ROIs, pMVS explained more variance than pReidsAv (the parameter determined from the angle of Reid’s plane). In the one remaining ROI, cerebellum right, the explained variance was similar (see Table 1 and Fig. 5).

The modulatory effect of pMVS was most substantial (in terms of R-squared effect size) in areas that are considered “lower” in the vestibular processing hierarchy and therefore, process information “nearer” to the original signal type in the vestibular periphery (end-organ), such as the cerebellar vermis. The effect decreased continuously towards central cortical vestibular processing areas, such as the anterior cingulum, and the left and right posterior insula.

The direction of the pMVS modulatory effect was dependent on both the side of the hemisphere and the level in the vestibular processing hierarchy. Categorically, the modulatory direction was opposed for the left and right cerebellar areas, while the left and right insula showed the same modulatory direction, however, with different strengths (see amplitude statistics below and Fig. 4).

**Fig. 3:**
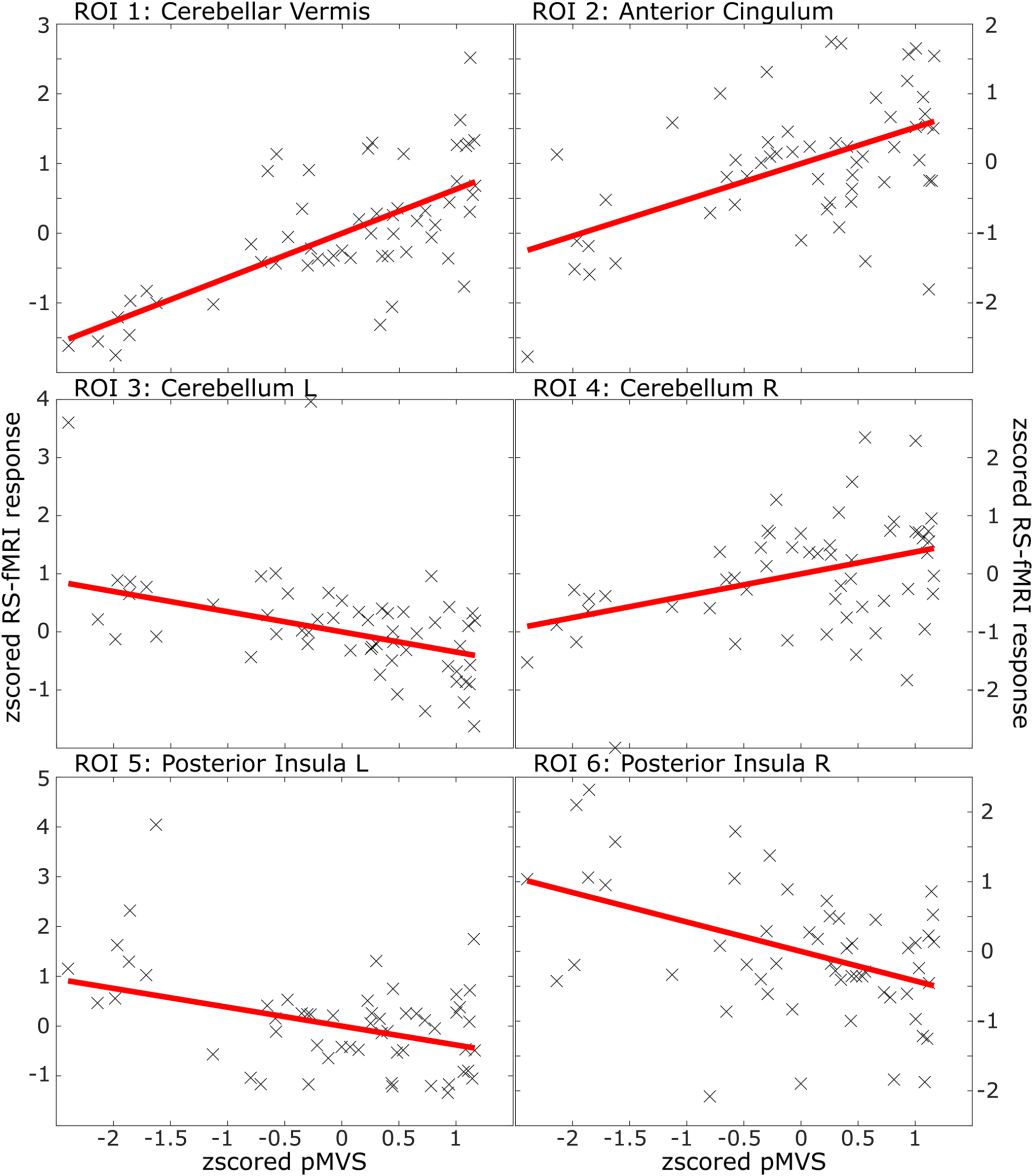
ROI- and subject-specific plot of the modulatory effect of the subject-specific proxy to account for MVS (or pMVS) on the RS-fMRI data after removal of the effects of all other regressors (age, time until the start of the RS-fMRI sequence, sex, handedness). Single-subject data were plotted as crosses. Trend lines in red show the fitted linear model for pMVS. Trends (verifiable in the slopes of the trend lines, see Fig. 4 and Table 1) were most reliable in areas that are considered “lower” in the vestibular processing hierarchy, such as ROI 1, cerebellar vermis. The effect decreased continuously towards central cortical vestibular processing areas, such as left and right posterior insula (ROI 5-6)

An ROI-specific overview of the average modulatory effect of pMVS on the resting-state data, with all data points of each participant plotted, can be seen in Fig. 3.

**Fig. 4:**
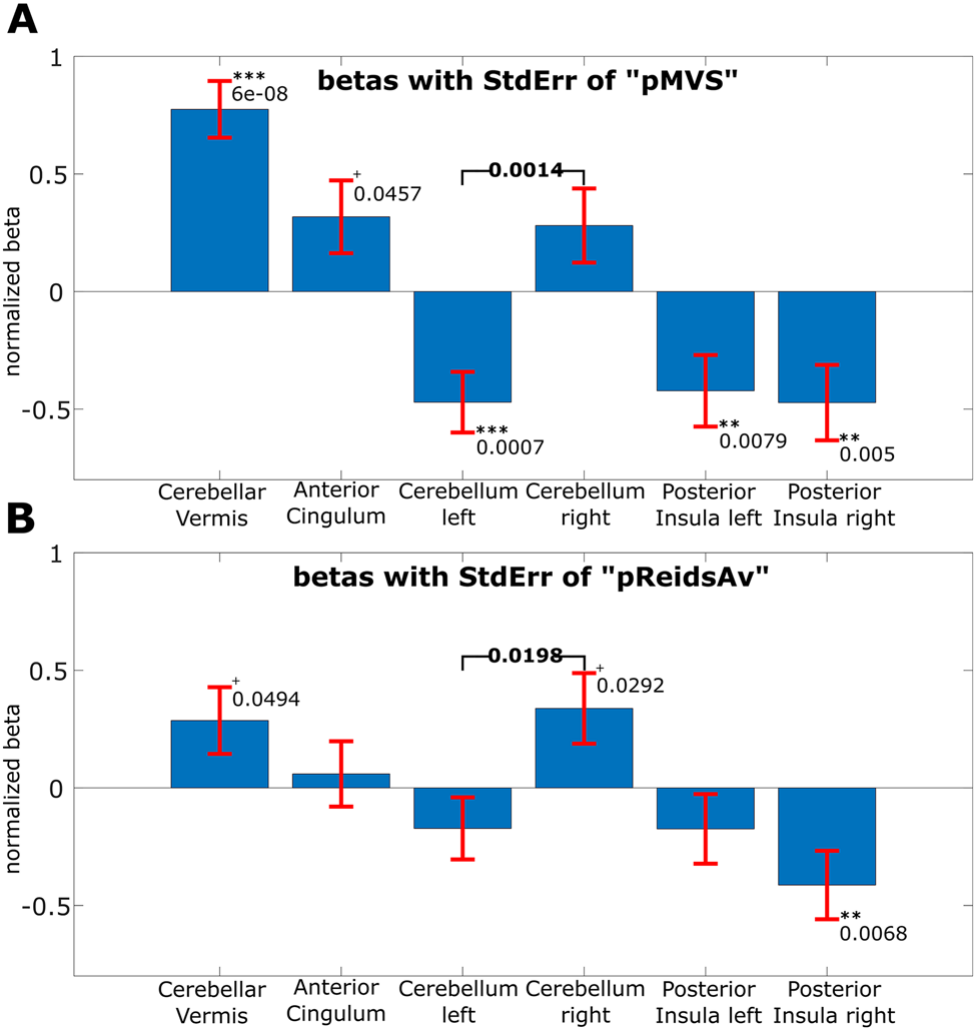
Bar graph of the estimated (normalized) slopes *β* for the effect of the subject-specific proxy to account for MVS, “pMVS” on top, labeled A, and for Reid’s plane at the bottom “pReidsAv”, labeled B. The red lines indicate the interval of twice the standard error of regression (SE), i.e., one SE above and one SE below the beta value. The slopes for left and right cerebellum (ROIs 3 and 4) showed a (statistically) significant difference, relative to each other (*p* = 0.0014 for “pMVS” and *p* = 0.0198 for “pReidsAv”), while the slopes for left and right posterior insula (ROIs 5 and 6) showed a slight but not (statistically) significant difference to each other. The slopes were estimated as normalized betas, i.e., the data and the regressors were z-scored before regression estimation. Therefore, each slope’s beta value indicated by how many standard deviations the RS-fMRI amplitudes will change for a one standard deviation change in pMVS or pReidsAv, respectively

The pMVS modulatory effect strength (the fitted slope *β* from the linear model varied between 0.774 for the cerebellar vermis ROI, and − 0.472 for the right posterior insula ROI, i.e., in the cerebellar vermis ROI a change of 0.744 standard deviations occurs per every one standard deviation change in pMVS. All betas for all regressors, their probability values for the test of the slopes against constant (H0: beta is zero, i.e., slope is flat), their standard error of regression (SE), and their individual added R-squared effect sizes are listed in Table 1.

The influence of pMVS on the left and right cerebellum ROIs was opposite (positive on the right and negative on the left) relative to each other (statistically significant: *p* < 0.0014; *d*Eff = 55 − 4 = 51). In contrast, the effect of pMVS on the left and right posterior insula had the same direction (negative, however, with only slightly different strengths (stronger for the right posterior insula, but not statistically significant: *p* = 0.26; *d*Eff = 55 − 4 = 51). For a depiction of the estimated slopes, *β* compare the bar graph in Fig. 4a. In brief, there are laterality differences in the cerebellum (statistically significant) and, to a lesser extent, also in the posterior insula (not statistically significant).

**Fig. 5:**
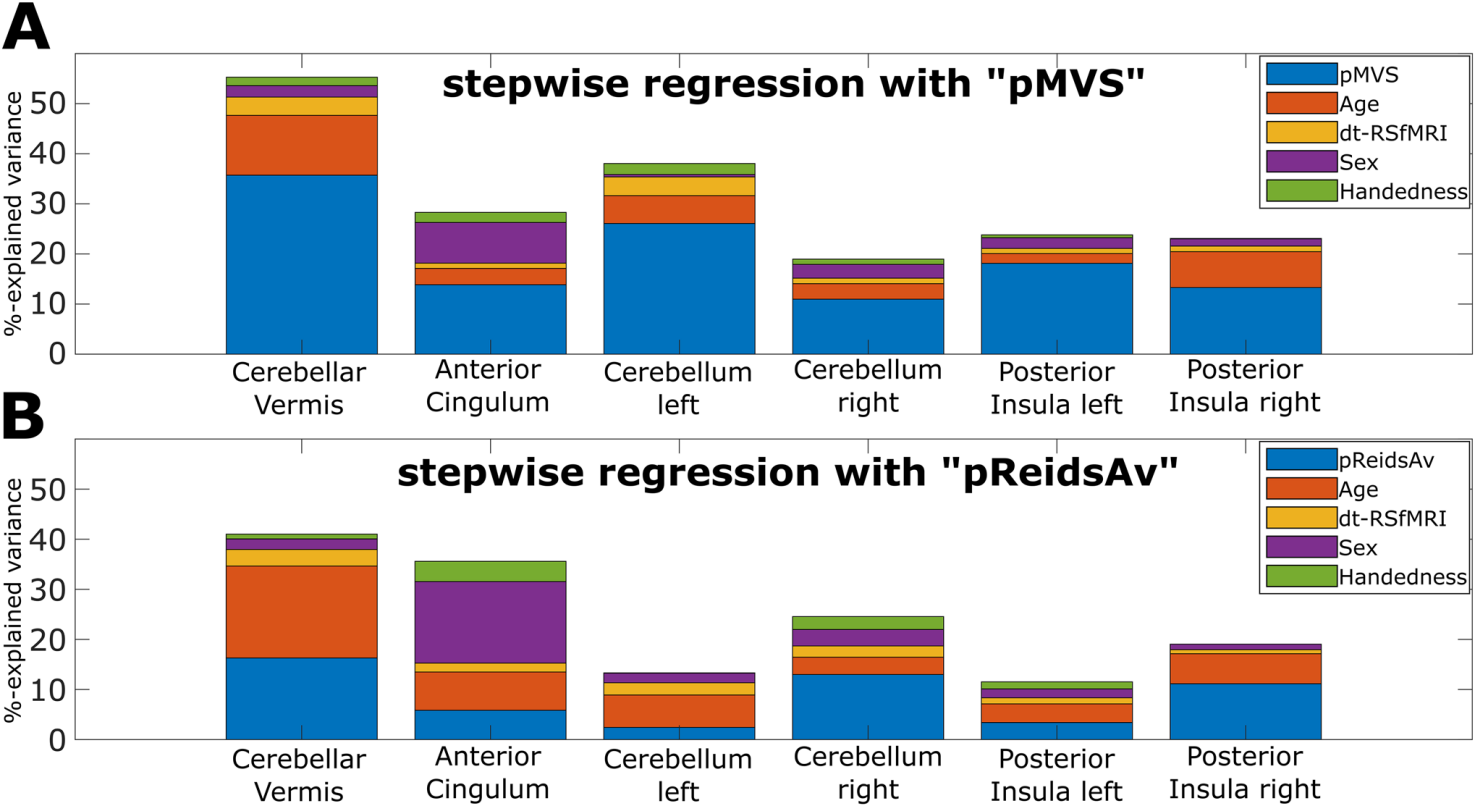
Depiction of the results of the stepwise regression model to determine the added explained variance (between subjects) for each variable in the regression model as stacked bar graphs. **a** The added explained variance between subjects, as a percentage of the total variance, for the model with “pMVS” and (**b**) the same model with “pReidsAv” instead of “pMVS”. Both graphs are for the same data and scaled in the same range 0–60% of explained variance. All the variables are listed in the top right corner

In all regions of interest, pMVS contributed the most substantial amount of the added explained variance (see Fig. 5). The explained variance for pMVS fluctuated between 11.0% for the right cerebellum (ROI 4), 13.3% for the right posterior insula (ROI 6), 13.9% for the anterior cingulum (ROI 2), 18.1% for the left posterior insula (ROI 5), 26.1% for the left cerebellum (ROI 3), and 35.7% for the cerebellar vermis (ROI 1).

All added variance per regressor and ROI are listed in Table 1 and displayed in Fig. 5.

## Discussion

This study aimed to examine the high between-subject variability in the modulatory effects of magnetic vestibular stimulation (MVS) on RS-fMRI fluctuations. As a result, the proxy for subject-specific orientation of the individual inner-ear anatomy relative to the direction of the static magnetic field (pMVS) was found to explain a large proportion of the between-subject variance in resting-state fMRI amplitudes (e.g., up to 42% in the cerebellar vermis). This parameter is easily implementable. An additional CISS sequence of the inner ear is the sole prerequisite. pMVS can then be added to group-level RS-fMRI analyses as a regressor to account for modulations due to MVS.

### Implications for the understanding of MVS

Following the Lorentz force model, a continuous ionic current within the inner ear exists between the dark cells and the utricular hair cells within the endolymph fluid independent of head movement or changing magnetic fields [3, 25]. Evoked by the magnetic field, a perpendicular Lorentz force pushes endolymph fluid onto the cupula in the horizontal (and superior) semicircular canal, which in turn leads to a shear of hair cells and thus to a change in the firing rate of the vestibular nerve, causing a state of vestibular imbalance [1–5, 25]. This model explains the occurrence, direction, and persistence of the observed nystagmus, but also its dependence on head positions within the magnetic field and the scaling relationship of RS-fMRI to the strength of the magnetic field itself [1–7]. The present results are following the proposed Lorentz force model for MVS as the proposed proxy to account for the subject-specific orientation of the individual inner-ear anatomy within the static magnetic field direction *(*pMVS) can be seen as a measure proportional to the Lorentz force that is at the basis of MVS [1, 3, 6].

### Implications for fMRI studies

Our results re-emphasize the importance of accounting for MVS when dealing with resting-state fMRI modulations. This becomes even more crucial with the usage of higher magnetic fields, and when investigating brain areas processing vestibular information or other sensory areas, especially of the visual system, that interacts with the vestibular system. The reason is that MVS modulation was shown to contribute to between-subject variance in a multiplicative manner with increasing field strength while the increase in the BOLD signal remains sublinear [4, 5]. Therefore, fMRI at higher field strength will increase between-subject variability disproportionately in vestibular areas or modulate areas that interact closely with the vestibular system, such as the visual system [20, 26–28], if MVS is not accounted for.

We recommend dealing with undesired MVS effects in fMRI pre- and post-data acquisition: Pre-data acquisition, MVS can be noticeably reduced by adjusting the head position of each subject until no nystagmus is detectable. This can be done by recording the eye movements of each subject during data acquisition via MR-compatible video-oculography (VOG) in total darkness [4]. However, this approach requires extra equipment (i.e., VOG) and know-how, since measuring eye movements while supine during MRI can be challenging and time consuming. Furthermore, it might not always be possible to adjust the head position until the nystagmus has disappeared, as the coils used in MRI can be restrictive to prevent head motion and, in this case, head reorientation. Therefore, we recommend adding a high-resolution structural sequence to the MR data acquisition protocol in order to be able to account for the orientation of the individual inner-ear anatomy in the static magnetic field, such as a “constructive interference steady state” (CISS) sequence. Post-data acquisition, the effects of MVS can then easily be accounted for by adding a regressor of no interest to any group-level analysis. This regressor can either be the single-subject nystagmus slow-phase velocity or the idiosyncratic morphology parameters of the inner ear *(*pMVS), as proposed in this study.

### Implications for vestibular research

MVS can also be considered a desirable tool that offers a novel and “cleaner” way of stimulating the vestibular system and creating controllable and adjustable vestibular imbalances. Furthermore, high-resolution imaging of the inner ear provides a proxy parameter (the sinus of the angle *α* between the horizontal SCC and the direction of the static magnetic field “*z*-direction”), which can account for the MVS differences between subjects in group-level analyses.

It was observed that the modulatory effect of pMVS seemed to be strongest in areas that are considered “lower” in the vestibular processing hierarchy and therefore process information “nearer” to the original signal type in the vestibular periphery end-organ) and decreased the nearer the area is to central cortical vestibular processing areas. This makes sense when considering that vestibular information is processed bilaterally and hierarchically on different levels according to their sensorimotor function: reflexive sensorimotor control of eyes, head, and body at the brain stem/cerebellar level; perception of self-motion and control of voluntary movement and balance at the cortical/subcortical level; and higher vestibular, cognitive functions (e.g., spatial memory and navigation) at the multisensory cortical level [9, 21, 29]. On its processing path from the periphery (end-organ) to the center (core area in the insular-opercular region, also referred to as parieto-insular vestibular cortex, PIVC), vestibular information already seems to be further processed from level to level. This might be the reason why MVS seems to show a decrease in the modulatory effect towards higher and more central areas. The effect was the weakest in the left and right posterior insula, which is considered a part of the vestibular core region within a network of multiple vestibular cortex areas in both hemispheres [9, 30–32]. This also fits well with the results of a previous combined clinical and modeling approach to understand different dizziness manifestations in acute unilateral midbrain lesions [33], where it was hypothesized that the different manifestations of dizziness according to the lesion level within the midbrain might be due a difference of vestibular cell populations. Midbrain strokes rarely presented with transient rotational vertigo and manifested with lesions chiefly in the caudal midbrain tegmentum, while the more frequent manifestations with swaying, unspecific, or no vertigo chiefly occurred in rostral mesencephalic or meso-diencephalic lesions. Animal experiments have shown that angular head-velocity cells are located mainly in the lower brainstem up to the midbrain, whereas the head direction cells are found from the midbrain and thalamic level up to cortical regions [33–38].

Other notable features of the vestibular system are a hemispheric asymmetry of the right non-dominant hemisphere in right handers and the left in left handers [20], and the dominance of the ipsilateral ascending projections on the side of the stimulated vestibular end-organ [39, 40]. In this regard, side differences in the influence of MVS on resting-state fluctuations were observable. The effects of MVS on the left and right side of the cerebellar regions of interest were opposite in direction. This might be due to different inputs from the periphery and the general fact that the cerebellum is connected to the integration of vestibular signals. The effects on the left and right side of the posterior insula have the same direction but are slightly different in their magnitude. Although not statistically significantly different, this might suggest that increasing variance due to MVS could obscure side differences, as seen in the response of the posterior insula to galvanic or caloric vestibular stimulation, especially when examined with fMRI. In line with this hypothesis, lateralization effects in the vestibular system were most substantial (apparent side differences of activations) in an experiment performed with PET imaging [20] without a magnetic field and could also be demonstrated in behavioral experiments [41]. In comparison, an fMRI experiment with the same setup showed less pronounced differences between the hemispheres and lateralization could only be established via differences in voxel cluster sizes for left handers and right handers, rather than apparent differences in activation for left handers and right handers [42]. Given our current results and previous studies under different magnetic field strengths, it seems possible that MVS has obscured lateralization effects in fMRI studies in the past [4, 5].

### Methodical limitations

There are methodical limitations in the current study that need to be considered in the interpretation of the data. First, we cannot measure current density in the inner ear, which is the second factor that can vary in the Lorentz force model besides the angle between the current and the magnetic field. Currently, we do not know how to measure this current strength non-invasively in humans. Was it possible to measure this current strength? And this would be a great additional tool for the study of MVS and the vestibular periphery in general.

Second, simultaneous VOG was not performed during the experiment to assess the MVS-associated slow-phase velocity of the induced nystagmus. Also, no segmentation of the resting-state sequence into times of “resting with interoception”, during which the MVS-associated nystagmus is present, and other times in which the nystagmus is suppressed and active exploratory eye movements are performed by the subjects, was not done. This could be an approach for future research into the dynamics of MVS in the individual subject.

Third, no conscious percept of the subjects, such as the strength of their vestibular percept and differences in alertness or how much they engaged in interoception, was assessed. A reporting system or questionnaire for assessing these values would be useful for further studies.

Fourth, we have not assessed the full morphometry of the inner ear. For example, the relationship of the utriculus position and orientation towards the horizontal and superior semicircular canals would be an interesting topic for future studies. We leave this and other assessments of the inner-ear morphology to future research when higher-resolution imaging and detailed morphometrical modeling are available.

Fifth, there was no systematic variation of the time until the RS-fMRI sequence was started, i.e., how long the subjects were in the MRI before the RS-fMRI sequence started. However, all subjects were in the MRI for several minutes previous to the RS-fMRI sequence, i.e., enough time for the MVS dynamic modulation effect to have stabilized [43–45]. The time difference was used as a regressor and the rank-transformed version was also included; both regressors did not explain a large fraction of the variance in this study. It could be an interesting topic for future studies to examine dynamic changes over the RS-fMRI acquisition within individual subjects concerning the dynamic changes in MVS in the first few minutes upon entering the MRI.

Sixth, we have focused on between-subject variance, and, likely, our suggestion for the simple regression of inner-ear morphology parameters is not sufficient enough to remove the MVS effect in single-subject RS-fMRI. In order to measure the full variability due to MVS in resting-state fMRI for a single subject, a study with subjects undergoing substantial head angle changes (and therefore change in the angle for the semicircular canals) would be needed in each subject with simultaneous eye movement recordings and fMRI acquisition for many different head positions repeated in multiple MRI B0 field strengths produced by different MRI scanners. This could then allow measurement of the MVS modulation effect on the individual level. In other words, one would have to not only do an experiment with different MRI B0 magnetic field strengths but also simultaneously observe the modulation due to different head orientations for a subject in such a way that the current density intrinsic to the subject remains the only determinable factor. Besides, future studies are needed to examine the influence of both vestibular end organs with all their separate components, such as left and right, anterior, posterior and horizontal semicircular canals as well as utricle and saccule, on the nystagmus as well as the different vestibular brain areas in RS-fMRI studies.

## Conclusion

Excess variability due to MVS should be addressed in an fMRI study analogous to nuisance regression for movement, pulsation, and respiration effects. MVS-induced additional between-subject variance can easily be accounted for by measuring the inner-ear morphology parameters of each subject and including these parameters in the group-level analysis.

## Abbreviations

MR: Magnetic resonance
B0: The static magnetic field used in MRI
MRI: Magnetic resonance imaging
FMRI: Functional magnetic resonance imaging
MVS: Magnetic vestibular stimulation
RS: Resting state
RSN: Resting-state network
L: Left
R: Right
RH: Right-handed
LH: Left-handed
SD: Standard deviation
SVV: Subjective visual vertical
